# Object Detection and Tracking with YOLO and the Sliding Innovation Filter

**DOI:** 10.3390/s24072107

**Published:** 2024-03-26

**Authors:** Alexander Moksyakov, Yuandi Wu, Stephen Andrew Gadsden, John Yawney, Mohammad AlShabi

**Affiliations:** 1 College of Engineering and Physical Sciences, University of Guelph, Guelph, ON N1G 2W1, Canada; 2 Department of Mechanical Engineering, McMaster University, Hamilton, ON L8S 4L8, Canada; wuy187@mcmaster.ca; 3 Adastra Corporation, Toronto, ON M5J 2J2, Canada; 4 Department of Mechanical and Nuclear Engineering, University of Sharjah, Sharjah P.O. Box 27272, United Arab Emirates; malshabi@sharjah.ac.ae

**Keywords:** estimation theory, Kalman filter, object detection, machine vision, sliding innovation filter, target tracking, YOLO

## Abstract

Object detection and tracking are pivotal tasks in machine learning, particularly within the domain of computer vision technologies. Despite significant advancements in object detection frameworks, challenges persist in real-world tracking scenarios, including object interactions, occlusions, and background interference. Many algorithms have been proposed to carry out such tasks; however, most struggle to perform well in the face of disturbances and uncertain environments. This research proposes a novel approach by integrating the You Only Look Once (YOLO) architecture for object detection with a robust filter for target tracking, addressing issues of disturbances and uncertainties. The YOLO architecture, known for its real-time object detection capabilities, is employed for initial object detection and centroid location. In combination with the detection framework, the sliding innovation filter, a novel robust filter, is implemented and postulated to improve tracking reliability in the face of disturbances. Specifically, the sliding innovation filter is implemented to enhance tracking performance by estimating the optimal centroid location in each frame and updating the object’s trajectory. Target tracking traditionally relies on estimation theory techniques like the Kalman filter, and the sliding innovation filter is introduced as a robust alternative particularly suitable for scenarios where a priori information about system dynamics and noise is limited. Experimental simulations in a surveillance scenario demonstrate that the sliding innovation filter-based tracking approach outperforms existing Kalman-based methods, especially in the presence of disturbances. In all, this research contributes a practical and effective approach to object detection and tracking, addressing challenges in real-world, dynamic environments. The comparative analysis with traditional filters provides practical insights, laying the groundwork for future work aimed at advancing multi-object detection and tracking capabilities in diverse applications.

## 1. Introduction

Object detection and tracking stand at the forefront of the most widely embraced domains in machine learning, given the limitless potential offered by various computer vision technologies. Numerous applications in autonomous robotics heavily rely on computer vision, representing the current singular approach to replicating human ocular functionality [1,2]. However, real-world tracking scenarios present considerable challenges, leading to potential degradation in tracking performance. These challenges encompass interactions among objects, occlusions, high degrees of similarity between distinct objects, background interference, and more [3]. Consequently, these challenges may result in undesirable outcomes such as bounding box drift or the misidentification of objects.

As the field of object detection continues to advance, the persistent challenge lies in achieving the most precise and reliable feature extractions from a given frame. Presently, object detection frameworks can be broadly categorized into two main groups: two-stage and one-stage detectors [4,5]. Two-stage detectors operate by generating a series of candidate regions that may contain objects, and then classifying each region and performing a bounding box regression based on the region’s features [6,7]. On the other hand, one-stage detectors do not identify candidate regions and instead utilize convolutional neural networks (CNN) to directly regress the location of and classify every object within the entire image [6,8,9,10].

In 2014, Girshik et al. introduced a Region-based Convolutional Neural Network (R-CNN) as an alternative to class deformable part models (DPMs), showcasing its superior performance on the PASCAL visual object classes (PASCAL VOC) dataset [11,12]. Despite its effectiveness, the R-CNN was hampered by its substantial computational demands. Addressing this limitation, He et al. proposed the Spatial Pyramid Pooling Network (SPP-Net) in 2015, which departed from the R-CNN’s approach by directly generating a feature map for the entire image and subsequently partitioning features for candidate regions. This innovation significantly enhanced both training and inference speed without compromising detection accuracy [13]. Girshik et al. subsequently proposed the Fast R-CNN, which uses a multi-task loss function and directly trains the CNN for classification and regression on two separate branches [14]. Despite proving to be more accurate than its predecessor, the Fast R-CNN required up to two seconds to detect an image on a typical operating machine. Ren et al. remedied this with the proposal of a Faster R-CNN, which utilizes the design of a region proposal network (RPN) that shares the convolution features of full images with the detection network [15]. The Faster R-CNN has achieved the top ranking on the PASCAL VOC dataset [12] and is acknowledged as the pioneering detection method that successfully implements end-to-end training. Despite the notable advancements made by two-stage detectors, the challenge of attaining real-time detection speed persists as a significant issue warranting further attention.

Redmon et al. proposed an efficient one-stage detector termed You Only Look Once (YOLO) in 2015 [16], which exhibited the capability to conduct real-time object detection. In contrast to the majority of two-stage detectors, YOLO omits the initial generation of proposed candidate regions. Rather, YOLO executes the regression and classification of all objects within an image simultaneously. Although the computational speed of YOLO far exceeds most of the existing state-of-the-art techniques, its detection accuracy was still inferior to that of the Fast R-CNN. The Single Shot Multibox Detector (SSD), introduced by Liu et al. [17], utilizes a network trained for the detection of objects across diverse scales, leveraging feature layers with varying depths. While SSD demonstrates detection speed similar to YOLO and accuracy on par with Faster R-CNN, it exhibits limitations in effectively detecting small objects. Redmon et al. subsequently proposed YOLOv2 in 2017, which makes use of the Darknet-19 CNN architecture to extract features from objects [18]. YOLOv2 displays an improvement in both detection accuracy and speed compared to the original YOLO architecture. YOLOv3 represents the conclusive iteration of the YOLO framework as proposed by Redmon et al. [19]. It incorporates a feature pyramid network and is founded on the enhanced Darknet-53 architecture. YOLOv3 improves upon the prior version through the introduction of a binary cross-entropy loss function, enhancing its ability to detect smaller objects and overall detection accuracy [20]. However, due to the inherent nature of the information fusion employed by YOLOv3, it is unable to make full use of low-level information, a weakness which has restricted its potential application in industry [20]. Bochkovskiy et al. proposed YOLOv4 [21], which uses CSPDarknet-53 as the backbone architecture. Additionally, it features a spatial pyramid pooling module, self-adversarial training, and employs genetic algorithms to optimize hyperparameter selection [20,22]. Overall, the YOLO framework has established itself as the state-of-the-art in object detection when evaluated on the MS COCO object detection benchmark dataset [21]. Furthermore, the framework has been widely applied in many industrial and practical applications and settings [23,24,25].

Target tracking is a distinct yet interconnected task, closely associated with the object detection challenge. Its primary objective is to anticipate the motion trajectory of an object following its initial detection over a defined duration. Traditionally, target tracking has been mostly carried out using estimation theory techniques, namely the Kalman filter (KF) [26,27,28,29,30]. The KF necessitates a linear mathematical model of the system or object under consideration, along with knowledge of its measurement and process noise characteristics. The KF also presupposes that both the process and measurement noise exhibit a zero-mean characteristic and adhere to a Gaussian distribution, commonly referred to as white noise. When these conditions are met, the KF provides the online optimal estimate of the states that minimize the mean square error [31,32,33,34]. Significant development effort has been directed towards the KF, with improvements to numerical stability, extensions to nonlinear systems, and many other enhancements [35,36].

In many real systems, *a priori* information about the underlying dynamics and noise is not known—or known only to a limited degree. Moreover, systems have the potential to undergo abrupt changes and exhibit new dynamic characteristics. This may arise either from inherent operation in multiple modes or as a result of unforeseen alterations in parameters or the occurrence of faults. The KF as well as other estimators can often struggle in these situations, as their underlying mathematical representation of the system is rendered inaccurate. The sliding innovation filter (SIF) is a sub-optimal, yet robust, estimation filter that has been recently proposed by Gadsden et al. [37]. The accuracy and computational complexity of the SIF have been shown to be superior compared to existing sub-optimal estimators like the smooth variable structure filter (SVSF), all while maintaining robustness to uncertainties and disturbances [37,38].

The objective of this research is to integrate the advanced object detection capabilities inherent in the YOLO architecture with the robust target tracking features offered by the SIF. Various efforts exploring the use of the KF with the YOLO architecture for combined object detection and tracking exist in the literature, especially since the KF is relatively simple to implement and tune effectively. Nevertheless, to the authors’ best knowledge, this is the first study to explore a more robust filter, specifically the novel SIF, for target tracking in machine vision applications. Moreover, this research endeavors to establish a benchmark for both the KF and the SIF, along with their nonlinear extensions—the Extended Kalman Filter (EKF) and Extended SIF (ESIF), respectively. Through a comparative analysis, this study seeks to provide insights into the performance of these filters within the domain of target tracking in machine vision applications.

The rest of this paper is organized as follows: Section 2 provides an exposition on the KF, SIF and their nonlinear extensions. In Section 3, a detailed overview of the proposed architecture is presented, including the experimental methodology and simulations to evaluate the proposed architecture. The results of the experiment are presented and discussed in Section 4, and Section 5 offers concluding remarks.

## 2. Estimation Theory

### 2.1. Kalman Filter

The KF is an estimator which provides the optimal solution for linear estimation problems. Considering a set of state and measurement equations as shown in Equations (1) and (2), the objective of an estimator is to determine the true value of the states $x_{k+1}$ from noisy measurements $z_{k+1}$ [39].

(1) $$x_{k+1}=Ax_{k}+Bu_{k}+w_{k},$$

(2) $$z_{k+1}=Cx_{k+1}+v_{k+1}.$$

In the equations above, $w_{k}$ and $v_{k}$ represent the system noise and measurement noise, respectively, and are generated using the noise covariance matrices *Q* and *R*, respectively. The KF operates under the assumption of zero-mean and Gaussian distributed system and measurement noises [35]. From above, *A* denotes the system matrix, *B* is the input gain matrix, and *C* is the measurement matrix. It is presupposed that matrices *A*, *B* and *C* remain fixed with respect to time.

Formulated as a predictor–corrector, the KF adheres to an iterative procedural framework [40]. The initial phase of this iterative process is the prediction stage, where the state estimates are calculated using the previous state values and knowledge of the system of interest’s dynamics, as demonstrated in Equation (3). Subsequently, the state error covariance is calculated according to Equation (4).

(3) $${\hat{x}}_{k+1|k}=A{\hat{x}}_{k|k}+u_{k},$$

(4) $$P_{k+1|k}=AP_{k|k}A^{T}+Q_{k}.$$

The update stage of the KF involves utilizing the state error covariance derived in Equation (4) from the prediction phase to update the innovation covariance $S_{k+1}$ and Kalman gain $K_{k+1}$ in Equations (5) and (6), respectively. The Kalman gain is employed to update the estimated state, as per Equation (7). The state error covariance is also updated using its previous value, as per Equation (8), which also concludes the first iteration of the process.

(5) $$S_{k+1}=C_{k+1}P_{k+1|k}C_{k+1}^{T}+R_{k+1},$$

(6) $$K_{k+1}=P_{k+1|k}C_{k+1}^{T}S_{k+1}^{-1},$$

(7) $${\hat{x}}_{k+1|k+1}={\hat{x}}_{k+1|k}+K_{k+1}\left(z_{k+1}-C_{k+1}{\hat{x}}_{k+1|k}\right),$$

(8) $$P_{k+1|k+1}=\left(I-K_{k+1}C_{k+1}\right){)P_{k+1|k}\left(I-K_{k+1}C_{k+1}\right))}^{T}+K_{k+1}R_{k+1|k}K_{k+1}^{T},$$

where *I* refers to the identity matrix with the dimensions of n×n, where *n* is the number of states. The subscripts *k* refer to the time step of the system, k|k refers to the updated values at the previous iteration, and k+1|k refers to the predicted values at time k+1. Additionally, it is noteworthy that in its traditional form, the KF assumes that the underlying system dynamics and measurement equations are linear. Thus, Equations (3)–(8) represent the process for linear systems and measurements only [37].

### 2.2. Extended Kalman Filter

The KF can be adapted for nonlinear systems, albeit with a compromise in optimality. The Extended Kalman Filter (EKF) employs a linearization technique: the first-order Taylor series expansion to approximate nonlinearities regarding the states of interest. This process of linearizing the nonlinearities is also known as the determining of Jacobian matrices. The resultant Jacobian matrix is subsequently used to update the state error covariance matrix. It is noted that in highly nonlinear systems, relying solely on a first-order Taylor series may lead to an imprecise approximation of the system’s behavior, and by extension, potential instabilities in the estimation process [31].

For a nonlinear system function $f({\hat{x}}_{k|k},u_{k})$ and nonlinear measurement function $h({\hat{x}}_{k|k})$. It is possible to derive linearized representations of these functions, commonly referred to as Jacobian matrices, through the following procedure:

(9) $$F_{k}={\left.\frac{\partial f(x)}{\partial x}\right|}_{x={\hat{x}}_{k|k},u_{k}},$$

(10) $$H_{k+1}={\left.\frac{\partial h(x)}{\partial x}\right|}_{x={\hat{x}}_{k+1|k}},$$

where $F_{k}$ refers to the linearized system matrix, $H_{k+1}$ is the linearized measurement matrix [37].

The EKF follows a similar process to the KF, and its prediction stage encompasses multiple procedural steps. Initially, the states undergo prediction through the utilization of Equation (11). Subsequently, the prediction of the state error covariance is executed per Equation (12), wherein the linearized system function is employed.

(11) $${\hat{x}}_{k+1|k}=f\left({\hat{x}}_{k|k},u_{k}\right),$$

(12) $$P_{k+1|k}=F_{k}P_{k|k}F_{k}^{T}+Q_{k}.$$

The equations governing the update stage of the EKF closely resemble those of the KF. The primary distinction lies in the utilization of the linearized measurement matrix $H_{k+1}$ in place of the conventional measurement matrix *C*. The linearization process involves approximating the system and measurement functions around the state estimate from the preceding time step. It is noted that due to the inherent approximation associated with linearization, the EKF no longer guarantees optimal state estimates [41].

(13) $$S_{k+1}=H_{k+1}P_{k+1|k}H_{k+1}^{T}+R_{k+1},$$

(14) $$K_{k+1}=P_{k+1|k}H_{k+1}^{T}S_{k+1}^{-1},$$

(15) $${\hat{x}}_{k+1|k+1}={\hat{x}}_{k+1|k}+K_{k+1}\left(z_{k+1}-h\left({\hat{x}}_{k+1|k}\right)\right),$$

(16) $$P_{k+1|k+1}=\left(w-K_{k+1}H_{k+1}\right))P_{k+1|k},$$

### 2.3. Sliding Innovation Filter

The SIF is a new filtering technique that, akin to the KF, is also formulated as a predictor–corrector. It is acknowledged as a sub-optimal filter, as it does not yield the optimal solution for the linear estimation problem, as detailed in prior works [42,43]. Nevertheless, the SIF incorporates sliding mode concepts and a switching gain, imparting inherent robustness in addressing challenges associated with ill-conditioned estimation problems, modeling uncertainties, and disturbances [44,45,46].

The prediction stage of the SIF closely parallels that of the KF’s and involves the prediction of state estimates ${\hat{x}}_{k+1|k}$, the state error covariance $P_{k+1|k}$ and innovation ${\tilde{z}}_{k+1|k}$ as follows:

(17) $${\hat{x}}_{k+1|k}=A{\hat{x}}_{k|k}+Bu_{k},$$

(18) $$P_{k+1|k}=AP_{k|k}A^{T}+Q_{k},$$

(19) $${\hat{z}}_{k+1|k}=z_{k+1}-C{\hat{x}}_{k+1|k}$$

The update stage involves the calculation of the SIF gain $K_{k+1}$, as well as the updated state estimates ${\hat{x}}_{k+1|k+1}$ and state error covariance $P_{k+1|k+1}$, per the three following equations, respectively:

(20) $$K_{k+1}=C^{+\bar{sat}}\frac{|{\tilde{z}}_{k+1|k}|}{\delta },$$

(21) $${\hat{x}}_{k+1|k+1}={\hat{x}}_{k+1|k}+K_{k+1}{\tilde{z}}_{k+1|k},$$

(22) $$P_{k+1|k+1}=\left(I-K_{k+1}C_{k+1}\right)P_{k+1|k}{\left(I-K_{k+1}C_{k+1}\right)}^{T}+K_{k+1}R_{k+1}K_{k+1}^{T},$$

where the $C^{+}$ term refers to the pseudoinverse of the measurement matrix, $\bar{sat}$ refers to the saturation term’s diagonal, sat refers to the saturation of a value (yielding a result between −1 and +1), $|{\tilde{z}}_{k+1|k}|$ refers to the absolute value of the innovation, and δ refers to the sliding boundary layer width.

**Figure 1:**
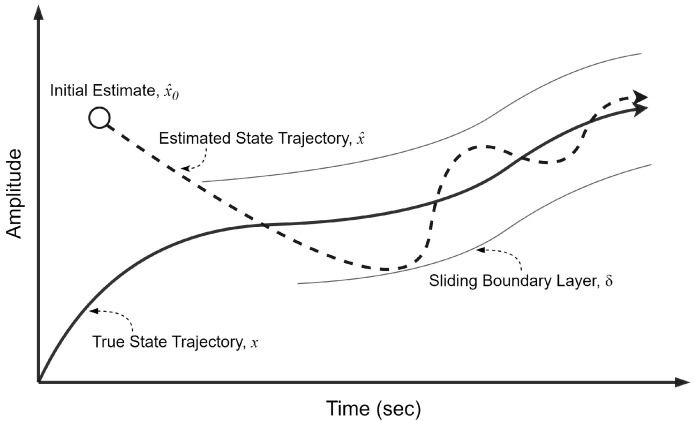
The sliding innovation filter and its sliding boundary layer [37].

The primary difference between the KF and the SIF lies in their gain structures. The KF’s gain is derived as a function of the state error covariance, which offers optimality [35]. As for the SIF, its gain is based on the measurement matrix, the innovation, as well as a sliding boundary layer term. Despite the state error covariance not being used in the calculation of the SIF gain, it is still beneficial in the sense that it provides useful information regarding the estimation error in the filtering process [37]. An overview of the SIF estimation concept is illustrated in Figure 1. The initial estimate is driven towards the sliding boundary layer, which is a parameter that is defined based on the degree of uncertainties in the estimation process. Once within the sliding boundary layer, the estimates are forced to switch about the true trajectory through the SIF gain [37].

### 2.4. Extended Sliding Innovation Filter

Similar to the KF and the EKF, the SIF can be extended to nonlinear systems by linearizing the system and measurement equations, yielding the Extended Sliding Innovation Filter (ESIF) [37]. The computation of the Jacobian matrices for these equations closely parallels the methodology employed in the EKF, as delineated in Equations (9) and (10). Otherwise, the structure of the ESIF estimation process aligns closely with that of the SIF, with the only difference manifesting in the formulation of the gain [37].

The ESIF’s prediction stage consists of three equations, with the initial two equations mirroring those of the EKF, as shown in Equations (11) and (12). The concluding equation in the prediction stage is expressed as follows:

(23) $${\tilde{z}}_{k+1|k}=z_{k+1}-h\left({\hat{x}}_{k+1|k}\right).$$

The update stage also encompasses three equations, the first of which is shown in the following Equation (24):

(24) $$K_{k+1}=H_{k+1}^{+\bar{\left\{sat\right\}}}-\frac{|{\tilde{z}}_{k+1|k}|}{\delta },$$

where $H_{k+1}^{+}$ refers to the pseudoinverse of the linearized measurement matrix at time k+1. The remaining two equations of the ESIF’s update stage remain unchanged and identical to the SIF’s update stage, as per Equations (21) and (22).

## 3. Proposed Strategy and Methodology

**Figure 2:**
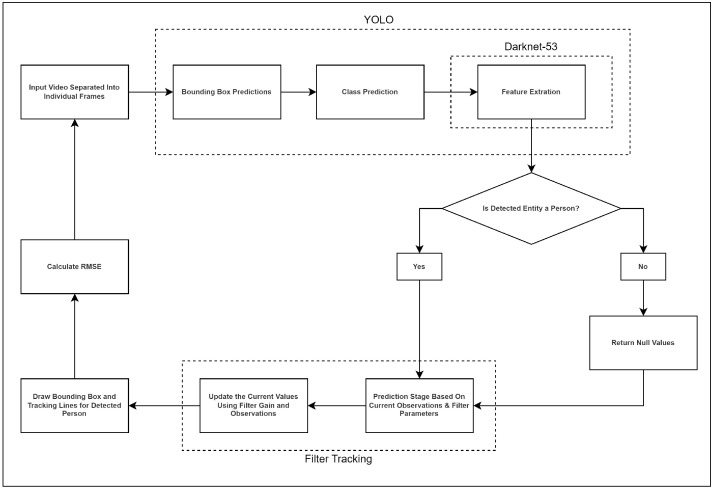
Flow diagram of proposed architecture and methodology.

A novel methodology is presented herein, aiming to leverage the YOLO framework for object detection while integrating it seamlessly with the tracking functionalities inherent in well-established estimation theory filters such as the KF and the recently proposed SIF. The envisaged architectural model is delineated into two primary stages, illustrated in Figure 2. The initial stage encompasses the application of the YOLO algorithm for efficient object detection, followed by the subsequent stage where the trajectories of identified objects are systematically tracked through the utilization of estimation theory techniques.

The initial phase of the proposed architecture involves the systematic processing of the video dataset through a sequential pipeline, resulting in the segmentation of the dataset into individual frames or images. These single-frame images are subsequently inputted into the YOLO framework, which is responsible for generating bounding boxes around identified objects of interest. The coordinates representing the center of these bounding boxes are then outputted as pairs of *X* and *Y* positional values and formulate the input to the subsequent filtering stage.

Next, upon confirmation that the object detected is of interest, the estimation filter under study (the KF, SIF, or their extended nonlinear extensions) undertakes the prediction stage, utilizing the previously obtained center coordinates from the YOLO framework. As previously mentioned, these input values are the *X* and *Y* values outputted in the previous step for each detected object. Through the subsequent prediction stage, the coordinates from the prior step for each detected object facilitate the assignment of a distinctive identification number to each object and the subsequent updating of their center coordinates and bounding boxes. The updated center coordinates and bounding boxes are compared to the true value of the frame, as processed and detected by the YOLO network, by calculating the root mean squared error (RMSE) value.

(25) $$RMSE=\sqrt{\frac{\sum_{i=1}^{n}y_{i}-{\hat{y}}_{i}}{n}}$$

The RMSE, as shown in Equation (25), is a widely employed metric in regression analysis, serving as a robust indicator of the predictive accuracy of a model. Mathematically, it is defined as the square root of the mean of the squared residuals, where residuals represent the discrepancies between the actual value ($y_{i}$) and its corresponding prediction (${\hat{y}}_{i}$), over total sample points available (*n*). The residual for each observation is squared to ensure that positive and negative deviations from the model predictions contribute meaningfully to the assessment. The mean squared error (MSE) is then computed by averaging these squared residuals over the entire dataset, and the final step involves taking the square root of the MSE to yield the RMSE. In essence, the RMSE provides a comprehensive measure of the dispersion of prediction errors, with lower values signifying a closer alignment between predicted and actual values. The determination of the RMSE value allows for benchmarking and comparing the performance of each of the estimation filters under scrutiny.

As discussed in earlier sections, various estimation theory techniques will be integrated with the YOLO architecture to facilitate object detection and tracking. As for the YOLO architecture utilized herein, a readily available model published by the creators of the architecture is employed. The model used is comprised of pre-trained weights from the MS COCO dataset, which allows for the ability to easily detect common objects such as humans, cars, airplanes, furniture, and many others [21]. Furthermore, the YOLO detection mechanism creates a bounding box around each detected object and displays the respective percent confidence for each object being correctly identified. Regardless, for the sake of effectively presenting the findings of this research, only bounding boxes around a singular object of interest are made visible.

The main filters under study for tracking the object of interest are the KF and SIF, as well as their nonlinear extensions the EKF and ESIF, respectively. The systems of interest are assumed to be of linear dynamics. The measurement function, state of the system and the vector of observations are all declared initially with zeros, and of size matching the expected shape of the images passed. Then, specific attributes are chosen, such as the uncertainty covariance, state transition matrix, as well as the process and state uncertainties. It should be noted that the process and state uncertainties may be tuned. For this study, empirical values were chosen for the mentioned parameters through a systematic grid selection tuning process to optimize the research outcomes.

**Figure 3:**
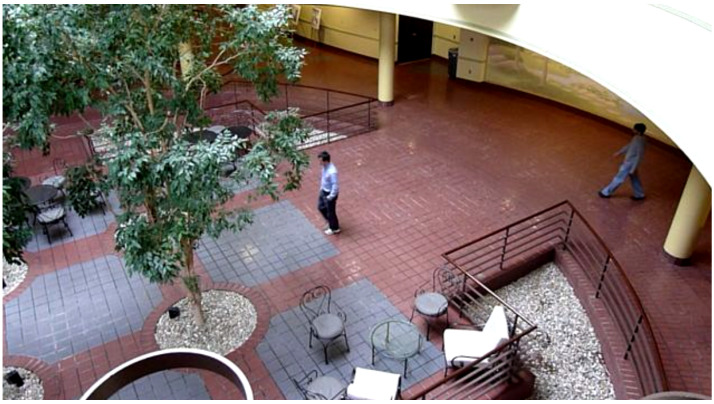
Still frame image from footage of the atrium dataset used in the experimentation.

The dataset used to carry out the simulations is stock footage consisting of a 15-s clip of students walking in an atrium, and a sample of a frame from the footage is shown in Figure 3. This dataset was chosen as it best replicates a closed-circuit television (CCTV) system, being that it is retrieved from a single-shot, stationary camera. Furthermore, the dataset footage involves multiple students within most of the frames, adding a degree of complexity to the proposed method. This complexity is useful in demonstrating the distinguishing capabilities of the proposed algorithm, especially considering that several objects of the same category may appear but are each individually unique.

**Figure 4:**
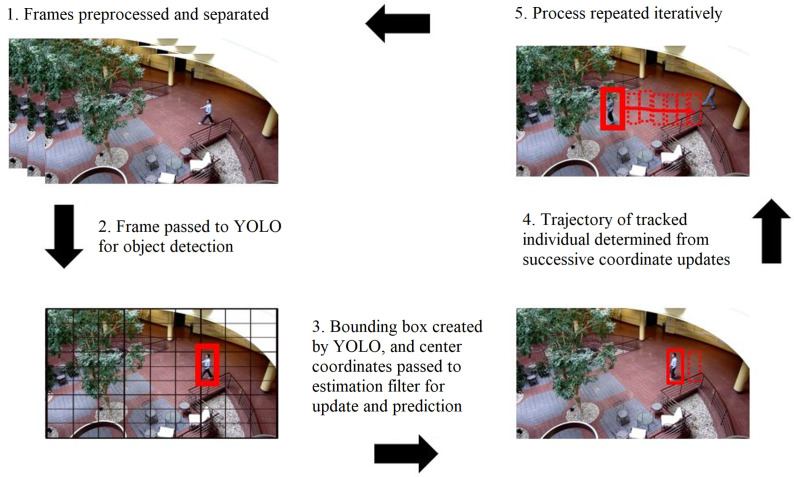
Object detection and tracking with YOLO and estimation theory for the dataset under study. The object of interest is the human within the bounding box.

The 15-s footage involved in the study resolves to 483 individual frames, each of which must be subjected to the proposed methodology. Thus, each individual frame or image will contain the bounding boxes and percent confidence of the detected object from the YOLO algorithm as well as the unique tracking trail of the object as determined by the filter in the study. The tracking trail has a unique color corresponding to the unique identity of each object and shows the entire trajectory from the entering frame up to the exiting frame. A schematic of the entire object detection and tracking process of the object of interest is illustrated in Figure 4.

Object detection and tracking often work with impeccable effectiveness when an accurate environment and domain are simulated for experimentation. However, in practical settings, tracking can often become difficult, especially for edge cases that were not anticipated or accounted for during development. One major problem that commonly arises is the problem of when the object that is being tracked is eclipsed by another larger object, and the tracker loses sight of the object of interest. This problem is encountered quite frequently within surveillance settings and applications, as the target can typically blend in with either a crowd of others or hide behind a building or other large objects. In crowd and human tracking scenarios, this is an especially critical problem when trying to follow the trajectory of specific individuals.

In response to the aforementioned issue, another experiment is conducted within this research to compare the effectiveness of the KF, EKF, SIF and EKF in handling situations where the video or tracking object may be interrupted. To simulate the problems that may arise in practical scenarios, interruptions are artificially injected into the video and into batches of frames to emulate the effect of a disturbance in our experimental simulations in the form of an occlusion. Specifically, three batches of five frames were interrupted at varying stages of the video near its beginning, middle, and end stages. For instance, shortly after the beginning of the video and after the filter under study has the chance to capture and track the moving object of interest, a batch of five frames would be removed from processing. This removal of frames essentially results in an interruption to the proposed architecture’s process. Upon the conclusion of the frame interruptions, the filter’s ability to reengage with the object of interest and resume tracking will be the main factor to be studied.

## 4. Results and Discussion

The results of the experiment involving object detection with YOLO and tracking with the different estimation filters such as the KF, EKF, SIF and ESIF are presented and discussed within this section of the paper. Each filter’s results will be discussed qualitatively by examining the trajectory produced for the individual that can be seen in the center of Figure 3. A quantitative analysis of each filter’s performance will also be carried out by examining the RMSE plots and computing the average RMSE of each filter’s tracking performance. The task required of the proposed method is to detect the first human to enter the scene and track their trajectory for the remainder of the simulation. Although the YOLO object detection framework may detect other individuals entering the scene, multi-object tracking is beyond the scope of this research, and thus, they will not be tracked by the filter under study.

### 4.1. Normal Conditions

**Figure 5:**
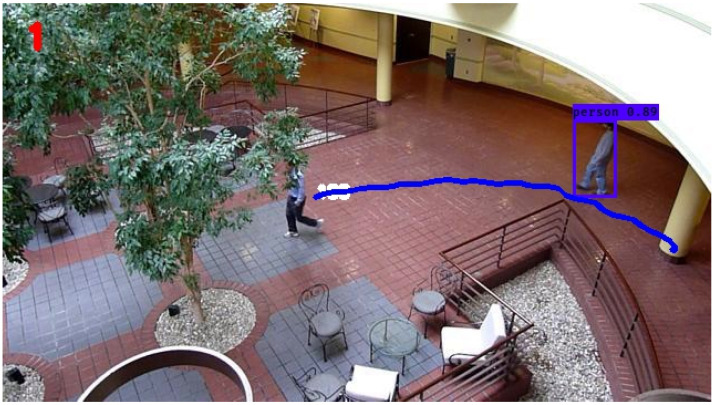
Still frame atrium image with Kalman filter tracking.

In the following simulations, normal operating conditions are maintained. The video dataset underwent preprocessing and was utilized in its original form, without any modifications to simulate disturbances. The performance of the KF in tracking the individual of interest’s trajectory, denoted by the blue line, can be seen in Figure 5. From this figure, it is evident that the KF was able to smoothly trace the individual’s movement from the right side to the left side of the atrium, with no apparent interruptions in the trajectory. The calculated average RMSE value for the KF over the entire simulation period was 1.34, serving as the established baseline for subsequent comparisons with other filters.

**Figure 6:**
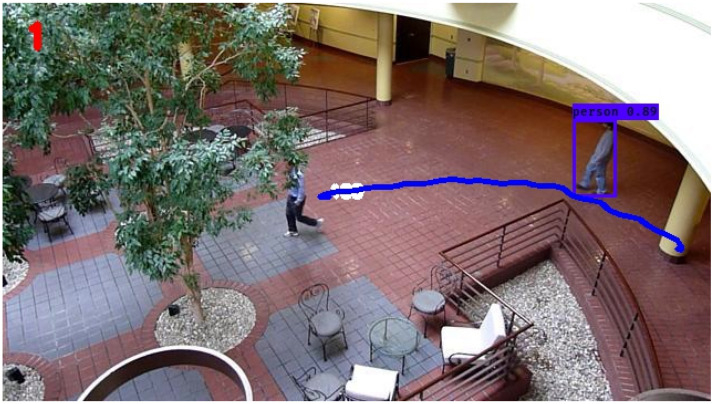
Still frame atrium image with extended Kalman filter tracking.

The trajectory determined by the nonlinear extension of the KF, the EKF, is displayed in Figure 6. Upon initial inspection, there are no conspicuous distinctions between the trajectories of the KF and the EKF. On closer examination, however, a notable artifact becomes apparent towards the final stages of the EKF’s determined trajectory. Specifically, a discernible perturbation is observed, leading to a loss of smoothness in the concluding frames of the trajectory when compared to the KF. Otherwise, no other major observations can be made qualitatively from the figure of the EKF’s trajectory. The average RMSE of the EKF across the entire simulation is determined to be 1.87, representing a 39.6 percent increase over the KF.

**Figure 7:**
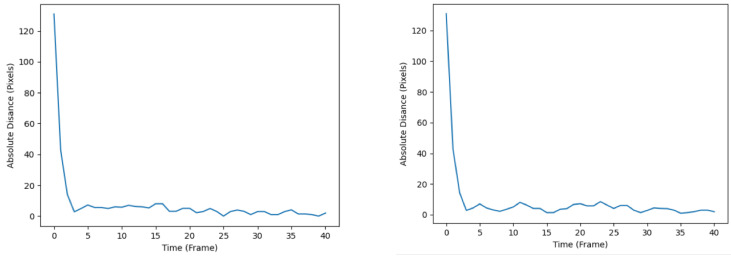
RMSE plots of the KF (**left**) and EKF (**right**).

The diagrams illustrating the RMSE progression for both the KF and EKF throughout the simulation are presented in Figure 7. The left subplot corresponds to the tracking performance of the KF, whereas the right subplot represents the tracking performance of the EKF. Notably, the RMSE plot associated with the KF demonstrates a comparatively smoother trajectory upon reaching its local optima. In contrast, the EKF’s RMSE plot exhibits more pronounced fluctuations, as evidenced by the heightened drops and rises after converging to the local optima. The observed disparities in tracking accuracy and effectiveness between the KF and EKF can be rationalized by considering the inherent characteristics of the filters. Specifically, the EKF, being a nonlinear filter, demonstrates inferior performance in tracking accuracy when applied to a system assumed to possess linear dynamics, as is the case in the present study. One can postulate that under the assumption of nonlinear system dynamics, the EKF is likely to perform better than the KF.

**Figure 8:**
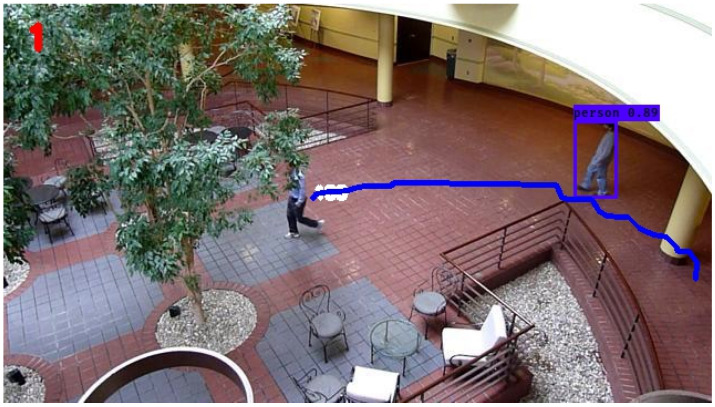
Still frame atrium image with sliding innovation filter tracking.

The subsequent filter under investigation is the SIF, and the trajectory associated with the tracked individual is depicted in Figure 8. Analysis of the figure reveals that the SIF generates a trajectory characterized by increased turbulence and reduced smoothness in comparison to both the KF and EKF. During the initial phases of the monitored individual’s path, the SIF exhibits erratic behavior, particularly when the individual changes course or direction. Subsequently, as the individual maintains a consistent trajectory, the SIF showcases the capacity to track smoothly, appearing to do so more seamlessly than both the KF and its nonlinear counterpart, the EKF. Overall, the SIF demonstrates an average RMSE value of 1.26, representing a 6 percent and 33 percent improvement over the KF and EKF, respectively.

**Figure 9:**
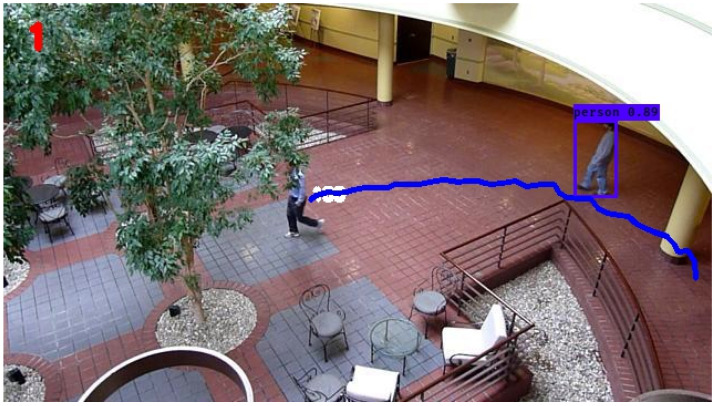
Still frame atrium image with extended sliding innovation filter tracking.

The trajectory determined by the ESIF is displayed in Figure 9. Similar to the SIF, the ESIF shows signs of volatile behavior in the earlier stages of the simulation, coinciding with the change in direction of the individual being tracked. In the latter segment of the simulation, the ESIF continues to accurately capture the trajectory of the individual of interest. However, during periods when the individual traverses without a change in direction, the ESIF manifests slight perturbations. In comparison to the SIF, the tracking capabilities of the ESIF do not manifest an equivalent level of smoothness. The average RMSE of the ESIF throughout the simulations is 1.33, which represents a negligible improvement over the KF. The SIF still represents an improvement of 5 percent in terms of RMSE compared to the ESIF.

**Figure 10:**
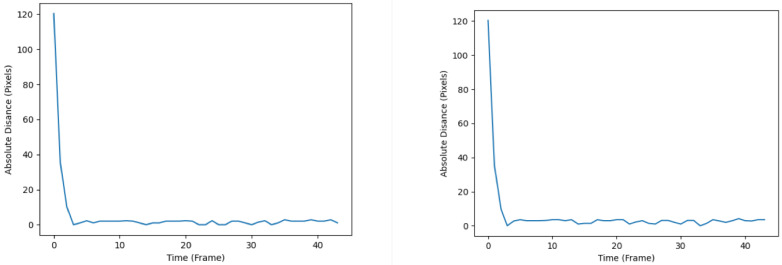
RMSE plots of the SIF (**left**) and ESIF (**right**).

The RMSE plots throughout the simulation are shown in Figure 10, whereby the left plot represents that of the SIF, and the right plot represents that of the ESIF. The SIF’s plot seems to display relatively less turbulent behavior upon reaching the local optima. This is apparent from the smaller number of spikes and troughs after the first 10 frames. As for the ESIF, the general trend of the RMSE plot is similar to that of the SIF’s, but with the added volatility observed by the higher number of jumps in the RMSE. When comparing the plots produced by the SIF and ESIF in Figure 10 to that of the KF and EKF’s in Figure 7, it can be observed that the former’s RMSE plots exhibit more stable behavior in the steady-state phase of the tracking, and are thus considered more favorable.

A summary of the average RMSE values for all the filters that were studied and examined throughout this research is shown in Table 1. This table confirms the fact that in ideal circumstances and simulation scenarios, the SIF displays the most effective performance in terms of tracking accuracy. Specifically, the SIF represents a six percent improvement over the KF, a thirty-three percent improvement compared to the EKF, and a five percent improvement over the ESIF. In Table 2, a summary of the time elapsed until the completion of the simulation for each of the studied filters is presented, highlighting their respective computational complexities. A Monte-Carlo simulation was conducted, consisting of 500 total executions of the experiment, and the values shown in Table 2 represent the average elapsed time for each filter. It is apparent from the results tabulated that the improved tracking performance of the SIF comes at a slight cost of computational complexity. More precisely, the SIF is 3.8 percent slower than the KF, whereas the ESIF represents a 5.7 percent increase in elapsed time compared to the EKF.

### 4.2. Disturbance Conditions

In this subsection, we examine the effectiveness of the proposed methodology under disturbance conditions. As delineated in preceding sections of this document, three sets of 20 frames each were deliberately interrupted at various points along the video sequence—namely, its inception, midpoint, and conclusion—to replicate the impact of disturbances. Therefore, a total of three simulations were conducted. The first simulation, where the interruption occurs at the beginning stages, is labelled as ‘break 1’. Similarly, the simulation with interruptions injected in the middle stages is labelled ‘break 2’, and for interruptions at the end stage, ‘break 3’. In ‘break 1’, frames are interrupted from the seventh frame up until the twelfth, whereas ‘break 2’ interrupts the frames from frame 14 to 19, and lastly, ‘break 3’ interrupts the frames from 20 to 25.

**Figure 11:**
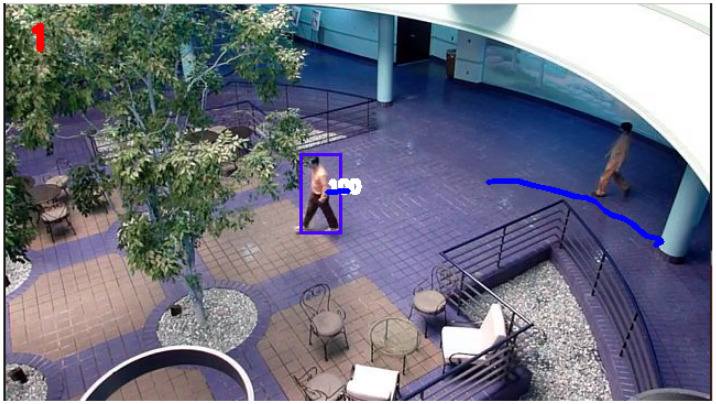
KF-based tracking performance under ‘break 3’ disturbance conditions.

**Figure 12:**
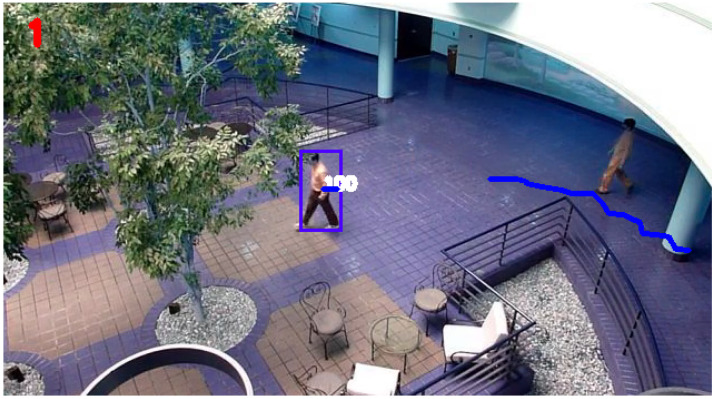
SIF-based tracking performance under ‘break 3’ disturbance conditions.

**Figure 13:**
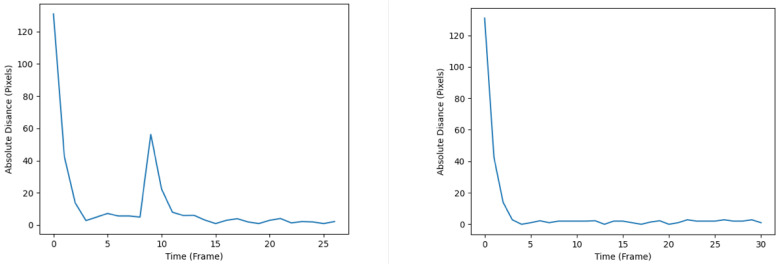
RMSE plots of the KF (**left**) and SIF (**right**) with the first break.

**Figure 14:**
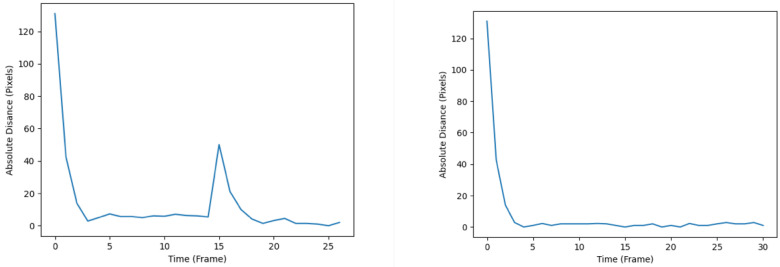
RMSE plots of the KF (**left**) and SIF (**right**) with the second break.

**Figure 15:**
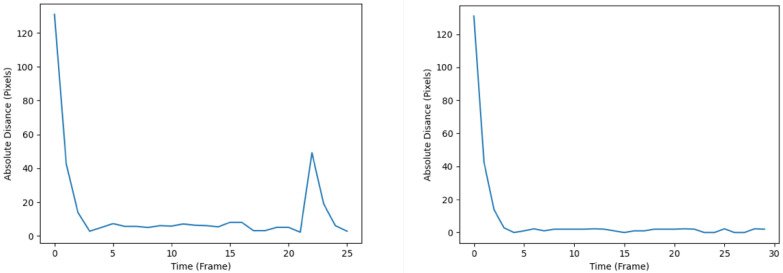
RMSE plots of the KF (**left**) and SIF (**right**) with the third break.

An example of the trajectory produced after ‘break 3’ disturbance conditions by the KF and SIF can be seen in Figure 11 and Figure 12, respectively. Otherwise, for the sake of brevity, only the respective RMSE plots for the KF and SIF under each break condition are shown, as can be seen from Figure 13, Figure 14 and Figure 15. From these plots, it can be seen that upon the injection of interrupted frames in the simulation, the KF reacts with a sudden spike in RMSE across all three trials. The spike in RMSE is non-trivial and represents an increase of approximately 300 percent, which subsides and returns to normal levels only upon the termination of the frame interruptions. Again, this observation is consistent across all three interruptions for the KF.

As for the SIF, from Figure 13, Figure 14 and Figure 15, it can be seen that despite the injected interruptions, there is no visible effect on the RMSE. Instead, the SIF can maintain effective tracking capabilities throughout the entire simulation, despite the three stages of interruptions throughout the simulation.

These observations are further confirmed when examining the average RMSE value for both the KF and SIF in each simulation, as can be seen in Table 3. From this table, it is evident that regardless of when the interruptions are injected into the simulation, the SIF is more robust in handling such disturbances and maintaining effective tracking performance. Across all three trials, the RMSE value of the SIF is less than half that of the KF. More specifically, the SIF represents a 45 percent RMSE reduction in ‘break 1’, a 42 percent reduction in ‘break 2’ and a 40 percent reduction in ‘break 3’.

### 4.3. Limitations

The proposed methodology, which integrates the sliding innovation filter with YOLO for real-time target tracking, exhibits several limitations that merit consideration. YOLO, renowned for its rapid object detection capabilities, faces an inherent challenge when coupled with the sliding innovation filter. Integrating the sliding innovation filter with YOLO introduces an additional layer of computational complexity to the algorithm. While YOLO excels in real-time object detection, the incorporation of a filtering mechanism like the sliding innovation filter amplifies the computational demands, potentially compromising the system’s real-time processing capabilities. The filter, designed to enhance target tracking precision, requires continuous adjustment and refinement based on evolving sensor measurements. This iterative process, although beneficial for refining predictions, adds an overhead to the existing computational load. Consequently, the amalgamation of YOLO and the sliding innovation filter presents a trade-off, as the pursuit of improved tracking accuracy through filtering introduces a computational burden that may impact the algorithm’s ability to operate seamlessly in real-time scenarios. The combined demands of both algorithms may lead to an increase in computational resources required, thereby impacting the system’s ability to sustain real-time performance. Furthermore, it is imperative to acknowledge the existence of alternative tools in the field, notably SORT and DeepSORT, which have established themselves in terms of efficiency and computational time. While our focus lies on presenting the innovative sliding innovation filter in conjunction with YOLO, it is essential to recognize the broader landscape of target tracking methodologies. A more comprehensive analysis, encompassing efficiency and computational time comparisons with existing tools, is a promising avenue for future research. Although not within the scope of the current work due to practical constraints, this acknowledgment paves the way for a more nuanced understanding of the strengths and limitations of various target-tracking approaches, thereby contributing to the ongoing discourse in the field.

### 4.4. Avenues of Future Research

In future work, we intend to broaden the scope of our evaluation by extending our experiments to widely accepted datasets and diverse scenarios. While the straightforward scenario in the current study allowed for a focused examination of the sliding innovation filter’s core functionality, the inclusion of more complex and varied scenarios will enable a comprehensive assessment of its performance across different tracking challenges. This extension will facilitate a more nuanced understanding of the method’s robustness and efficacy in real-world applications. Furthermore, recognizing the critical role that parameter optimization plays in the overall performance of tracking systems, our future research will delve into a detailed investigation of parameter tuning for both the sliding innovation filter and the YOLO algorithm. Although our current study aimed to demonstrate the effectiveness of the sliding innovation filter within the chosen context, we acknowledge the importance of fine-tuning parameters to enhance the system’s adaptability and performance across diverse scenarios. Additionally, in line with the need for a broader perspective, we plan to conduct a thorough comparison of our proposed approach utilizing the sliding innovation filter with existing tracking tools such as SORT and DeepSORT. This comparative analysis will specifically focus on efficiency and computational time, aiming to provide a comprehensive understanding of the relative strengths and weaknesses of each method. This analysis will contribute valuable insights to the field, assisting researchers and practitioners in selecting the most suitable tracking solution based on their specific requirements and constraints.

## 5. Conclusions

In this paper, a new framework for object detection and tracking using the YOLO architecture and novel estimation theory techniques has been proposed. The proposed method is based on the YOLO network, which can detect and classify objects and delimit the regions of interest such that their centroids can be calculated. Tracking of the objects is generally carried out by the Kalman filter, and in this work, we propose the use of the novel, more robust SIF. The experimental simulations carried out in this study demonstrate that in surveillance applications, the SIF is more effective in tracking the trajectory of subjects of interest that are detected by the YOLO network, especially in the presence of disturbances. When disturbances are artificially injected throughout the simulations, the SIF’s switching gain mechanism can maintain the true states within the sliding boundary layer, thus limiting their divergence from the true states. The SIF’s tracking advantage comes at a slight cost of computational complexity which can be considered negligible compared to the performance improvement. Further research efforts toward more careful tuning of the sliding boundary layer width may result in the realization of improved tracking performance. Furthermore, future efforts will be directed towards a reliable multi-object detection and tracking approach with the proposed methodology.

## Figures and Tables

**Table 1:** Average RMSE of various estimation strategies for human target tracking.

Estimation Strategy	RMSE
KF	1.34
EKF	1.87
SIF	1.26
ESIF	1.33

**Table 2:** Tabulated elapsed computation time of various filters.

Estimation Strategy	Time (s)
KF	19.1365
EKF	19.5899
SIF	19.8792
ESIF	20.7122

**Table 3:** RMSE of the KF and SIF subject to different breaks.

Break	KF	SIF
1	2.8892	1.2950
2	2.9414	1.2304
3	3.0778	1.2230

## Data Availability

Dataset available on request from the authors.
